# Preoperative Expectations and Postoperative Outcomes of Visual Functioning among Cataract Patients in Urban Southern China

**DOI:** 10.1371/journal.pone.0169844

**Published:** 2017-01-09

**Authors:** Ziyan Chen, Xianchai Lin, Bo Qu, Wuyou Gao, Yajing Zuo, Wenyan Peng, Ling Jin, Minbin Yu, Ecosse Lamoureux

**Affiliations:** 1 State Key Laboratory of Ophthalmology, Zhongshan Ophthalmic Center, Sun Yat-sen University, Guangzhou, China; 2 Singapore Eye Research Institute, Singapore National Eye Centre, Singapore; 3 Duke-National University of Singapore Medical School, Singapore; 4 Centre for Eye Research Australia, University of Melbourne, Melbourne, Australia; Soochow University Medical College, CHINA

## Abstract

**Purpose:**

To investigate the relationship between preoperative expectations and actual postoperative outcomes of visual function (VF) among patients undergoing first eye cataract surgery.

**Methods:**

A longitudinal study of 182 patients from hospitals in urban Southern China were surveyed prior to surgery and 3 month after cataract surgery regarding their preoperative, expected postoperative and actual postoperative VF for each of the items on the Catquest-9SF and their satisfaction with cataract surgery. In addition, detailed clinical data were collected preoperatively and postoperatively.

**Results:**

The majority of cataract patients in urban Southern China had high expectations for VF outcomes after cataract surgery and in most cases postoperative outcomes achieved the expected level of improvement. The mean (standard deviation, SD) preoperative Catquest-9SF score was 15.7 (5.86) and the mean (SD) expected postoperative score was 26.3 (2.93). The discrepancy between actual and expected improvement was significantly correlated with patients’ health literacy, presence of systemic and ocular comorbidity, preoperative visual acuity of the surgery eye, LOCS III nuclear opalescence and cortical cataract grading.

**Conclusion:**

Cataract patients in urban Southern China had high expectations for surgery outcomes. Patients with low level of health literacy and the presence of systemic and ocular comorbidity may need a comprehensive counseling to decrease the discrepancy regarding expected and actual outcomes.

## Introduction

Cataract remains the leading cause of blindness and visual impairment in China[1, 2]. The growing elderly population and advancements in medical technology have led to an increasing demand for cataract surgery[3, 4] which in turn substantially increases functional vision and vision-related quality of life[5–7]. Recent advances in the developments of safe, effective and simplified techniques in cataract surgery have increased the demand for surgery and visual outcome expectations[8, 9]. Recent data showed the 5-year incidence of cataract surgery in urban Southern China has improved, due to improved health care access[1]. In urban China, most cataract patients (89.6%) were willing to pay for cataract surgery although the costs for cataract surgery were higher than the average monthly income[10].

The patient-reported outcome and patient’s satisfaction, reflected by postoperative visual outcome achieving the preoperative expectation, has become an important indicator for the success of cataract surgery outcomes[9, 11]. Nevertheless, little information about preoperative patient expectations and postoperative patient satisfaction regarding the outcomes of cataract surgery is available in China, in which access, costs and quality of health care are changing rapidly.

Given the important role of vision function (VF) in daily life in elderly population, we aim to investigate the relationship between patient expectations and actual outcomes of VF after cataract surgery. In order to describe patients’ self-assessed outcome after ophthalmic interventions in a more comprehensive way, numerous questionnaires have been developed, and these include VF-12[8], VF-14[11] and VF-15[12]. The nine-item short-form Rasch-scaled Catquest-9SF was reliable and valid in measuring visual disability outcomes of cataract surgery[13–15], and has been validated previously in China[16]. In this study, we used Catquest-9SF questionnaire for patients undergoing the first eye cataract surgery in urban Southern China.

## Methods

### Patients

The study included patients age 18 years or older awaiting first eye cataract surgery at the Second Affiliated Hospital and Sun Yat-sen University, Guangzhou, Southern China between Jan 2014 and Jun 2014. All patients completed the Catquest-9SF questionnaire preoperatively and again approximately 3 months after cataract surgery and an additional questionnaire for information about patients' ophthalmic and medical histories, demographic characteristics, health literacy and computer skill. The clinical information was collected by the examining ophthalmologist. Written informed consent was obtained from all participants. The study adhered to the Declaration of Helsinki and was conducted after obtaining ethical approval by the Zhongshan Ophthalmic Center Institutional Review Board.

### Catquest-9SF questionnaire

Details regarding the Chinese version of the Catquest-9SF questionnaire has been described previously[16]. The Catquest-9SF questionnaire consisted of 7 questions for performing daily-life activities and 2 global questions about difficulties in general and satisfaction with vision. For the perceived difficulty levels, the response options were as follows: 1 = very great difficulty; 2 = great difficulty; 3 = some difficulty; 4 = no difficulty. For satisfaction with vision, the response options were as follows: 1 = very dissatisfied; 2 = rather dissatisfied; 3 = fairly satisfied; 4 = very satisfied. Lower scores generally indicate worse VF.

The method to define patient expectations regarding the outcome of cataract surgery on VF referred to the previous study[8]. If the patients, who reported any difficulty on the Catquest-9SF tasks, considered there were chance of an improvement after surgery, the patients were asked to estimate how much improvement she or he expected (a little, a moderate amount, a great deal or do not know). Based on these responses, we calculated an expected postoperative Catquest-9SF score for each patient. This was done by adjusting each preoperative item-specific score upward by the amount of expected postoperative improvement (a little = 1; a moderate amount = 2, a great deal = 3). If an expected score exceed 4, it would be recorded as 4. The summary score was calculated for 7 items.

### Questionnaire for health literacy, computer skills and demographic variables

For health literacy and computer skills measured by an interviewer-administered questionnaire were described previously[17]. Health literacy and computer skills were assessed by asking 3 questions. For health literacy, summary scores of > 10 and ≤10 were classified as inadequate and adequate, respectively. For computer skills, patients who answered ‘‘yes” to all questions were considered to have adequate computer skills. Otherwise, patients were classified as having inadequate computer skills.

### Clinical examination

Each patient underwent comprehensive eye examinations before and 3 months after cataract surgery. In addition to completion of the Catquest-9SF questionnaire, visual acuity, Lens Opacities Classification System (LOCS) III grading of cataract, and the type of implanted intraocular lens were recorded for each eye.

As introduced previously[18, 19], the LOCS III cataract severity was assessed under pupil dilation and graded for each of the 4 possible components: nuclear opalescence, nuclear color, cortical cataract, and posterior subcapsular cataract. For each item, higher grading scores indicate greater severity. The scale ranges from 0.1 (clear or colorless) to 5.9 (very opaque in cases of cortical and posterior subcapsular cataract) or 6.9 (very opaque in cases of opalescence and nuclear color). Patients underwent standard phacoemulsification cataract surgery and intraocular lens (IOL) implantation.

### Statistical methods

Only the surgical eye was included in the whole analysis. The main outcomes of the study were discrepancy between actual and expected postoperative Catquest-9SF scores (actual—expected) and the satisfaction with the surgery. Baseline data for demographic, health, ocular and visual functional characteristics of the patients were presented as mean (SD) for continuous variables with normal distribution and frequency (percentage) for categorical variables. Data for preoperative difficulty, expected and actual postoperative improvement on the Catquest-9SF items by demographic, ocular and functional characteristics were presented as median (Inter quartile range, IQR). The Wilcoxon signed rank sum test was used for comparing the difference between actual and expected postoperative Catquest-9 SF score by each level of the variables. The Kruskal Wallis test for variables with more than two levels and Wilcoxon-Mann Whitney test for variables with two levels were used for comparing the overall difference across groups of the variables between actual and expected postoperative Catquest-9 SF score. Chi-square test or Fisher’s exact test (one or more than one cell frequency < = 5) was used to compare the proportion of the patients achieving expected improvement between groups. Spearman's rank correlation coefficients were calculated for measuring the strength of the association between functional characteristics and preoperative, expected and actual postoperative Catquest-9SF scores. Linear regression and ordinal logistic regression analyses were performed for the discrepancy between actual and expected postoperative Catquest-9SF scores and the satisfaction with the surgery respectively. Variables with P<0.05 in the simple regressions with one predictor in the model were included in the multiple regression analysis with more than one predictors in the model. All statistical analyses were performed using a commercially available software package (Stata 13.1, StataCorp, College Station TX, USA).

## Results

Among 215 eligible patients, 182 were enrolled. Among these, 174 (95.6%) completed the detail preoperative and postoperative data and examination in the study. **Table 1**lists the demographic, health, ocular visual functional characteristics of the study population. Patients included in the analysis had a mean (SD) age of 71.0 (11.6) years (range 21.0–90.0 years). 51.7% were male and 52.0% of patients had low level of education. A total of 72 (39.6%) had inadequate healthy literacy, 179 (98.4%) had inadequate computer skills. 45.6% of patients had hypertension, diabetes or others, while 29.7% had coexistent glaucoma, age-related macular degeneration (AMD), diabetic retinopathy (DR) or others. 51.7% of cataract patients had an extremely poor visual acuity of 6/120 or worse before cataract surgery. 57.5% had a moderate or severe degree of LOCSIII grading NO (nuclear opalescence) score, 50.8% had a moderate or severe degree of LOCSIII grading C (cortical cataract) score. In 59.6% the regular cataract extraction with the implantation of aspherical IOL was performed. 60.3% reported a moderate amount or a great deal of trouble with vision and 88.5% were dissatisfied or very dissatisfied with preoperative vision. The mean (SD) expected postoperative Catquest-9SF score was 26.3 (2.93) (range 12–28), which was much higher (the difference by paired t test was 10.6, 95% confidence interval: 9.82, 11.3, P<0.001) than the mean (SD) preoperative Catquest-9SF score 15.7 (5.86) (range 7–28).

**Table 1:** Baseline demographic, health, ocular and visual functional characteristics of the participants had cataract surgery in only one eye (N = 182).

Characteristics	N	n (%)
**Demographic Characteristics**		
**Age (Year)**	175	
≤60		25 (14.3)
60–70		51 (29.1)
70–80		65 (37.1)
>80		34 (19.4)
Mean (SD), range		71.0 (11.6), (21–90)
**Sex**	182	
Male		94 (51.7)
Female		88 (48.3)
**Education**	173	
Primary school or lower		90 (52.0)
Junior school or higher		83 (48.0)
**Health literacy**	182	
Inadequate		72 (39.6)
Adequate		110 (60.4)
**Computer skill**	182	
Inadequate		179 (98.4)
Adequate		3 (1.60)
**Systemic comorbidity**		
Hypertension	182	27 (14.8)
Diabetes	182	35 (19.2)
Others	182	30 (16.5)
Any of the above	182	83 (45.6)
No	182	99 (54.4)
**Ocular comorbidity other than cataract**		
Glaucoma	182	5 (2.75)
AMD	182	3 (1.65)
Diabetic retinopathy	182	14 (7.69)
Others	182	34 (18.7)
Any of the above	182	54 (29.7)
No	182	128 (70.3)
**Ocular characteristics**		
**Presenting visual acuity of the surgery eye**	174	
＞6/60		45 (25.9)
＞6/120 - ≤6/60		39 (22.4)
≤6/120		90 (51.7)
**LOCS III Grading**		
**NO**	179	
≤3		76 (42.5)
＞3 - ≤5		80 (44.7)
＞5		23 (12.8)
**C**	181	
≤3		89 (49.2)
＞3 - ≤4		57 (31.5)
＞4		35 (19.3)
**IOL type**	161	
Aspherical IOL		96 (59.6)
Spherical IOL		65 (40.4)
**Visual functional characteristics**		
**Trouble with vision**	179	
Very difficult		51 (28.5)
Moderately difficult		57 (31.8)
Little difficult		62 (34.6)
Not difficult		9 (5.03)
**Satisfaction with vision**	182	
Very dissatisfied		89 (48.9)
Dissatisfied		72 (39.6)
Satisfied		17 (9.34)
Very satisfied		4 (2.20)
Preoperative Catquest-9SF score*		
Mean (SD), range	182	15.7 (5.86), (7–28)
Expected postoperative Catquest-9SF score**		
Mean (SD), range	182	26.3 (2.93), (12–28)

SD = Standard Deviation AMD = Age-related macular degeneration LOCS = Lens opacities classification system.

NO = Nuclear opalescence C = Cortical cataract IOL = Intraocular lens.

* The preoperative Catquest-9SF score was the total score of each preoperative item-specific score with range from 7 to 28.

** The expected postoperative Catquest-9SF score was calculated by adjusting each preoperative item-specific score upward by the amount of expected postoperative improvement coded as 1 = a little improvement, 2 = moderate improvement and 3 = great improvement. The maximum expected postoperative score for each item was limited to 4 and the range of the total expected postoperative score was 7–28.

**Tables 2**and **3**show the preoperative difficulty, expected and actual postoperative improvement on the Catquest-9SF items by demographics and ocular characteristics. The median expected and actual postoperative Catquest-9SF scores were high, regardless of patient characteristics such as age, gender, education, health literacy, computer skill and presence of any systemic disease (**Table 2**) and ocular characteristics such as preoperative visual acuity of the surgery eye, presence of any other ocular comorbidity, LOCS III grading and the type of implanted IOL (**Table 3**). For the most part, the median difference between actual and expected postoperative scores was significantly zero, indicating the expected improvement was achieved. In some subgroups, the median actual postoperative score was lower than the expected postoperative score. Patients with inadequate healthy literacy, diabetes, preoperative visual acuity worse than 6/120, any ocular comorbidity (diabetic retinopathy), mild LOCS III NO and C grading and the implantation of aspherical IOL had a significantly larger difference between actual and expected postoperative Catquest-9SF scores.

**Table 2:** Preoperative difficulty, expected and actual postoperative improvement on the Catquest-9SF items by demographics (N = 174)§.

		Catquest-9SF score Median (IQR)				Subjects achieving expected improvement n (%)¶
Characteristics	Number of subjects	Preoperative	Expected postoperative	Actual postoperative†	Difference of postoperative (Actual—Expected)‡	
**Age (Year)**						
≤60	25	17 (14–21)	28 (24–28)	28 (24–28)	0 (-2–1)	15 (60.0)
61–70	49	16 (11–20)	28 (25–28)	26 (24–28) *	-1 (-3–0)	23 (46.9)
71–80	62	14.5 (10–19)	28 (27–28)	27 (24–28) *	0 (-3–0)	35 (56.5)
＞80	31	14 (10–18)	28 (24–28)	27 (25–28)	0 (-2–2)	18 (58.1)
**Sex**						
Male	89	17 (13–21)	28 (27–28)	28 (25–28)	0 (-2–0)	44 (49.4)
Female	85	14 (10–20)	28 (24–28)	26 (23–28) *	-1 (-3–0)	51 (60.0)
**Education**						
Primary school or lower	86	16 (12–21)	28 (25–28)	27 (24–28)	0 (-3–0)	46 (53.5)
Junior school or higher	79	16 (11–20)	28 (25–28)	27 (25–28) *	0 (-2–0)	45 (57.0)
**Health literacy**						
Inadequate	68	14 (9–18)	28 (24–28)	25 (21.5–28)**	-1 (-3–1)	30 (44.1)
Adequate	106	18 (13–21)	28 (26–28)	28 (25–28)	0 (-2–0)	65 (61.3) *
**Computer skill**						
Inadequate	171	15 (11–21)	28 (25–28)	27 (24–28)**	0 (-3–0)	94 (55.0)
Adequate	3	18 (16–25)	28 (28–28)	27 (26–28)	-1 (-2–0)	1 (33.3)
**Systemic comorbidity**						
**Hypertension**						
Yes	26	12.5 (7–20)	26 (21–28)	24 (21–26)	-2 (-3–0)	11 (42.3)
No	148	16 (12.5–21)	28 (25.5–28)	27 (25–28)*	0 (-2–0)	84 (56.8)
**Diabetes**						
Yes	34	14.5 (10–19)	28 (25–28)	25 (21–27)	-1.5 (-4–0)*	13 (39.4)
No	140	16 (11.5–21)	28 (25–28)	27 (25–28)***	0 (-2–1)	81 (57.9)
**Any systemic comorbidity**						
Yes	80	14 (8.5–19.5)	26 (24.5–28)	26 (24–28)**	0 (-3–0)	41 (51.3)
No	94	18 (14–21)	28 (26–28)	28 (25–28)	0 (-3–1)	54 (57.5)

IQR = Inter Quartile Range AMD = Age-related macular degeneration.

§ Among 182 who had surgery, 174 (95.6%) persons had complete pre and post operative Catquest-9 SF data.

†The Wilcoxon signed rank sum test was used for comparing the difference between actual and expected postoperative Catquest-9 SF score by each level of the variables. P values were noted as *P<0.05, **P<0.01, *** P<0.001.

**‡**The Kruskal Wallis test for variables with more than two levels and Wilcoxon-Mann Whitney test for variables with two levels were used for comparing the overall difference across groups of the variables between actual and expected postoperative Catquest-9 SF score. P values were noted as *P<0.05, **P<0.01, *** P<0.001.

¶ The expected improvement was achieved if actual post operative Catquest-9 SF score was equal to or more than expected postoperative score. Chi-square test or Fisher’s exact test (one or more than one cell frequency < = 5) was used to compare the proportion of the subjects achieving expected improvement between groups. P values were noted as *P<0.05, **P<0.01, *** P<0.001.

**Table 3:** Preoperative difficulty, expected and actual postoperative improvement on the Catquest-9SF items by ocular characteristics (N = 174)§.

		Catquest-9SF score Median (IQR)				Subjects achieving expected improvement n (%))¶
Characteristics	Number of subjects	Preoperative	Expected postoperative	Actual postoperative†	Difference of postoperative (Actual—Expected)‡	
**Presenting visual acuity of the surgery eye**						
>6/60	41	18 (14–21)	28 (25–28)	28 (26–28)	0 (-1–2)	27 (65.9)
>6/120 - ≤6/60	39	17 (9–21)	28 (25–28)	28 (24–28)	0 (-3–0)	23 (59.0)
≤6/120	87	14 (10–19)	28 (25–28)	25 (23–28)***	-1 (-3–1)*	41 (47.1)
**Ocular comorbidity**						
**Glaucoma**						
Yes	5	14 (14–18)	28 (28–28)	23 (21–25)	-3 (-7–0)	2 (40.0)
No	169	16 (11–21)	28 (25–28)	27 (24–28)**	0 (-2–0)	93 (55.0)
**AMD**						
Yes	3	14 (10–16)	24 (22–28)	23 (21–28)	-3 (-5–6)	1 (33.3)
No	171	16 (11–21)	28 (25–28)	27 (24–28)**	0 (-3–0)	94 (55.0)
**Diabetic retinopathy**						
Yes	14	13 (9–16)	27.5 (23–28)	21 (21–25)**	-2.5 (-6–0)**	4 (28.6)
No	160	16 (12–21)	28 (25–28)	27 (24.5–28)*	0 (-2–0)	91 (56.9)
**Any ocular comorbidity**						
Yes	51	14 (12–19)	28 (25–28)	25 (21–28)***	-1 (-4–0)***	19 (38.0)**
No	123	17 (11–21)	28 (25–28)	28 (25–28)	0 (-2–1)	75 (61.5)
**LOCS III Grading**						
**NO**						
≤3	75	18 (14–21)	28 (25–28)	25 (23–28)**	-1 (-3–0)	36 (48.0)
>3 - ≤5	77	14 (8–21)	28 (25–28)	27 (25–28)	0 (-2–0)	45 (58.4)
>5	20	15 (9–18.5)	28 (27.5–28)	28 (25.5–28)	0 (-2–0)	12 (60.0)
**C**						
≤3	86	17 (14–21)	28 (25–28)	26 (23–28)**	-1 (-3–0)	42 (48.8)
>3 - ≤4	54	16 (11–21)	28 (27–28)	26.5 (24–28)*	0 (-2–0)	31 (57.4)
>4	33	11 (7–18)	28 (25–28)	28 (26–28)	0 (-1–1)	21 (63.6)
**IOL type**						
Aspherical IOL	94	15 (12–20)	28 (24–28)	25 (22–28)**	-0.5 (-3–0)	47 (50.0)
Spherical IOL	59	17 (11–21)	28 (28–28)	28 (27–28)	0 (-1–0)	34 (57.6)

IQR = Inter Quartile Range LOCS = Lens opacities classification system NO = Nuclear opalescence C = Cortical cataract IOL = Intraocular lens.

§ Among 182 who had surgery, 174 (95.6%) persons had complete pre and post operative Catquest-9 SF data.

†The Wilcoxon signed rank sum test was used for comparing the difference between actual and expected postoperative Catquest-9 SF score by each level of the variables. P values were noted as *P<0.05, **P<0.01, *** P<0.001.

**‡**The Kruskal Wallis test for variables with more than two levels and Wilcoxon-Mann Whitney test for variables with two levels were used for comparing the overall difference across groups of the variables between actual and expected postoperative Catquest-9 SF score. P values were noted as *P<0.05, **P<0.01, *** P<0.001.

¶ The expected improvement was achieved if actual post operative Catquest-9 SF score was equal to or more than expected postoperative score. Chi-square test or Fisher’s exact test (one or more than one cell frequency < = 5) was used to compare the proportion of the subjects achieving expected improvement between groups. P values were noted as *P<0.05, **P<0.01, *** P<0.001.

**Table 4**shows the preoperative, expected and actual postoperative Catquest-9SF scores by the level of patient reported trouble and satisfaction with vision. Patients with better vision had higher preoperative VF scores (Spearman correlation, 0.53; *P*<0.001), higher expected postoperative scores (Spearman correlation, 0.23; *P* = 0.002), higher actual postoperative scores (Spearman correlation, 0.68; *P*<0.001) and larger difference between actual and expected postoperative scores (Spearman correlation, -0.26; *P*<0.001). The level of satisfaction with vision was significantly correlated with the preoperative score (Spearman correlation, 0.21; *P* = 0.005) and actual postoperative score (Spearman correlation, 0.65; *P*<0.001), but not correlated with the expected postoperative score and the difference between actual and expected postoperative scores.

**Table 4:** Preoperative difficulty, expected and actual postoperative improvement on the Catquest-9SF items by functional characteristics (N = 174)*.

		Catquest-9SF score Median (IQR)					Subjects achieving expected improvement n (%)†§
Characteristics	Number of subjects	Preoperative†	Expected postoperative†	Actual postoperative†	Actual postoperative‡	Difference of postoperative (Actual—Expected)†	
**Self-report trouble with vision**							
Very difficult	49	11 (7–15)	28 (22–28)	28 (25–28)	/	0 (-1–2)	36 (73.5)
Moderately difficult	53	14 (11–18)	28 (25–28)	26 (24–28)	16.5 (10–23)	-1 (-3–0)	25 (47.2)
Little difficult	19	19 (16–21)	28 (27–28)	26.5 (24–28)	22 (21–24)	-1 (-3–0)	26 (43.3)
Not difficult	9	24 (20–28)	28 (28–28)	28 (26–28)	28 (26–28)	0 (-2–0)	5 (55.6)
Spearman correlation (P-value)		**0.53 (<0.001)**	**0.23 (0.002)**	-0.07 (0.35)	**0.68 (<0.001)**	**-0.26 (<0.001)**	/
**Self-report satisfaction with vision**							
Very dissatisfied	83	14 (8–19)	28 (25–28)	26 (24–28)	26.5 (25–28)	0 (-3–0)	43 (51.8)
Dissatisfied	70	18 (14–21)	28 (27–28)	27 (25–28)	21.5 (21–23)	0 (-3–0)	37 (52.9)
Satisfied	17	15 (11–18)	26 (24–28)	28 (25–28)	25 (21–26)	0 (-1–1)	12 (70.6)
Very satisfied	4	16 (14–19.5)	28 (26.5–28)	28 (24.5–28)	28 (27–28)	0 (-3.5–1.5)	3 (75.0)
Spearman correlation (P-value)		**0.21 (0.005)**	0.02 (0.80)	0.13 (0.09)	**0.65 (<0.001)**	0.09 (0.26)	/

*Among 182 who had surgery, 174 (95.6%) persons had complete pre and post operative Catquest-9 SF data.

**†** In this column, trouble and satisfaction with vision was collected before surgery.

**‡** In this column, trouble and satisfaction with vision was collected after surgery.

§ The expected improvement was achieved if actual post operative Catquest-9 SF score was equal to or more than expected postoperative score.

**Table 5**shows the expected and actual postoperative improvement in specific items of the Catquest-9SF. The percentage of patients who had difficulty in each activity before surgery ranged from 81.0% to 94.8%. The portion of patients who expected to improve in activities with difficulty before surgery ranged from 82.1% to 95.8%. The proportion of patients who reported improvement after surgery among those with difficulty before surgery ranged from 86.1% to 91.8%. The ratio of patients who achieved expected level of improvement ranged from 70.1% to 81.3% for each activity. The portion of preoperative difficulty tended to be higher in near vision dependent activities, e.g., ability to read newspaper, read prices when shopping and do needlework. And the ratio of who achieved expected level of improvement tended to be lower, among these activities for which the near vision is required.

**Table 5:** Expected and actual improvement in activities on the Catquest-9SF items (N = 174).

	% With difficulty before surgery	% With difficulty before surgery who expected to improve	% Who reported improvement after surgery among those with difficulty before surgery	% Who achieved expected level of improvement among those with difficulty before surgery
**Read newspaper**	94.8	95.8	86.1	70.3
**Recognize faces**	86.2	86.9	88.7	79.3
**Prices when shopping**	90.2	91.1	89.8	70.1
**Walk on uneven ground**	86.2	86.9	90.0	81.3
**Needlework**	89.1	90.0	89.7	74.2
**Seeing text on TV**	90.8	91.7	91.8	76.6
**Hobbies**	81.0	82.1	90.1	80.9

**Table 6**shows linear regression analysis of potential predictors of the difference between actual and expected postoperative overall Catquest-9SF scores. Simple linear regression analysis showed adequate health literacy (*P* = 0.04), presence of systemic comorbidity (diabetes) (*P* = 0.009), any ocular comorbidity other than cataract (*P*<0.001), preoperative visual acuity of the surgery eye (≤6/120) (*P* = 0.008) and LOCS III grading C score (>4) (*P* = 0.04) were the significant predictors. The multiple analysis also showed presence of systemic comorbidity (diabetes) (*P* = 0.04) and any ocular comorbidity other than cataract (*P* = 0.03) were the significant predictors. Patients with adequate health literacy had proper expectation for the postoperative VF outcomes which could be fulfilled. However, patients with poor prognostic indicators (such as combined systemic or ocular diseases, poor preoperative visual acuity and severe degree of cataract) had larger difference between actual and expected improvement of VF.

**Table 6:** Linear regression of potential predictors of the discrepancy between actual and expected postoperative Catquest-9SF scores (N = 174).

	Simple Regression		Multiple Regression*	
	β (95% CI)	P-value	β (95% CI)	P-value
**Age (Year)**	-0.008 (-0.06, 0.04)	0.75		
**Male sex**	0.58 (-0.48, 1.65)	0.28		
**Junior school or higher (versus primary school or lower)**	-0.06 (-1.16, 1.03)	0.91		
**Adequate health literacy**	**1.12 (0.03, 2.20)**	**0.04**	0.64 (-0.52, 1.79)	0.28
**Adequate computer skill**	-0.44 (-4.55. 3.67)	0.83		
**Hypertension**	-1.00 (-2.50, 0.49)	0.19		
**Diabetes**	**-1.78 (-3.10, -0.46)**	**0.009**	**-1.40 (-2.75, -0.05)**	**0.04**
**Any ocular comorbidity other than cataract**	**-2.05 (-3.19, -0.92)**	**<0.001**	**-1.38 (-2.63, -0.14)**	**0.03**
**Presenting visual acuity of the surgery eye**				
>6/60	Reference		Reference	
>6/120 - ≤6/60	-1.02 (-2.57, 0.53)	0.20	-0.87 (-2.41, 0.68)	0.27
≤6/120	**-1.79 (-3.11, -0.48)**	**0.008**	-0.98 (-2.33, 0.37)	0.15
**LOCS III Grading**				
**NO**				
≤3	Reference			
>3 - ≤5	0.90 (-0.25, 2.04)	0.12		
>5	0.50 (-1.27, 2.28)	0.58		
**C**				
≤3	Reference		Reference	
>3 - ≤4	0.17 (-1.04, 1.38)	0.78	-0.07 (-1.29, 1.15)	0.91
>4	**1.51 (0.08, 2.94)**	**0.04**	1.05 (-0.40, 2.49)	0.15
**IOL type**				
Aspherical IOL	-0.90 (-2.05, 0.24)	0.12		
Spherical IOL	Reference			

AMD = Age-related macular degeneration LOCS = Lens opacities classification system.

NO = Nuclear opalescence C = Cortical cataract IOL = Intraocular lens.

* Variables with P<0.05 in simple regression were included in multiple regression analysis.

**Table 7**shows the ordinal logistic regression analysis of potential predictors of the satisfaction with the surgery. The simple regression analysis showed patient satisfaction with the surgery was significantly associated with that presence of systemic comorbidity (hypertension) (*P* = 0.004), any ocular comorbidity other than cataract (*P*<0.001), LOCS III grading NO score (>3 to ≤5) (*P* = 0.03), LOCS III grading C score (>4) (*P* = 0.04) and the type of implanted IOL (*P* = 0.008). In the multiple analysis, presence of any ocular comorbidity (*P* = 0.007) appeared the major factor influencing patients' satisfaction with the cataract surgery.

**Table 7:** Ordinal logistic regression of potential predictors of the satisfaction with the surgery* (N = 172).

	Simple Regression		Multiple Regression**	
	OR (95% CI)	P-value	OR (95% CI)	P-value
**Age (Year)**	0.98 (0.95, 1.01)	0.14		
**Male sex**	0.50 (0.22, 1.12)	0.09		
**Junior school or higher (versus primary school or lower)**	0.96 (0.43, 2.18)	0.93		
**Adequate health literacy**	0.47 (0.22, 1.02)	0.06		
**Adequate computer skill**	2.17 (0.20, 23.5)	0.53		
**Hypertension**	**3.87 (1.56, 9.61)**	**0.004**	2.45 (0.91, 6.58)	0.07
**Diabetes**	1.89 (0.78, 4.57)	0.16		
**Any ocular comorbidity other than cataract**	**4.47 (1.99, 10.0)**	**<0.001**	**3.28 (1.38, 7.79)**	**0.007**
**Presenting visual acuity of the surgery eye**				
>6/60	Reference		Reference	
>6/120 - ≤6/60	2.22 (0.51, 9.57)	0.29		
≤6/120	3.35 (0.93, 12.1)	0.06		
**LOCS III Grading**				
**NO**				
≤3	Reference		Reference	
>3 - ≤5	**0.38 (0.16, 0.90)**	**0.03**	0.28 (0.07, 1.15)	0.08
>5	0.31 (0.07, 1.43)	0.13	0.29 (0.04, 2.12)	0.22
**C**				
≤3	Reference			
>3 - ≤4	0.90 (0.39, 2.07)	0.80	2.24 (0.57, 8.81)	0.25
>4	**0.12 (0.02, 0.92)**	**0.04**	0.54 (0.04, 6.48)	0.63
**IOL type**				
Aspherical IOL	**4.00 (1.43, 11.0)**	**0.008**		
Spherical IOL	Reference			

AMD = Age-related macular degeneration LOCS = Lens opacities classification system.

NO = Nuclear opalescence C = Cortical cataract IOL = Intraocular lens.

* Satisfaction with the surgery was coded as 1 = satisfied to 5 = very dissatisfied.

** Variables with P<0.05 in simple regression were included in multiple regression analysis. IOL type was not included in the multiple regression due to collinearity with NO (0.68) and C (0.61).

## Discussion

Using the Catquest-9SF questionnaire, we showed that the postoperative VF outcomes after cataract surgery achieved the expected level of improvement in the majority of cataract patients in urban Southern China. Our data showed that the discrepancy between actual and expected improvement was significantly associated with patient health literacy, presence of systemic and ocular comorbidity, preoperative visual acuity of the surgery eye and the degree of lens opacity, which was also associated with patient satisfaction with cataract surgery.

Our findings are consistent with those from previous studies showing that patients' preoperative expectations were high, irrespective of the demographic or ocular characteristics. The achievement of expectations, which is considered as the discrepancy between actual and expected improvement, varied in different studies[8, 9, 11, 12]. Tielsch et al. in 1991 showed that 61% of cataract patients (a total of 552 included) in the United States achieved or surpassed their expected level of postoperative VF at 4-month after cataract surgery using the VF-12[8]. Pager in Australia showed 66% of patients (a total of 121 included) failed to equal or exceed their expectations at 1-month after cataract surgery using the VF-14 and the expectation-outcome discrepancy correlated significantly with satisfaction[11]. Addisu et al. used the VF-15 questionnaire to find that the actual outcome of cataract patients in Ethiopia was marginally better than expected improvement and the degree of improvement was not significantly related to patient satisfaction at all[12].

We have shown in an earlier study, that Chinese elderly patients with cataract had inadequate health literacy and computer skills, which were significantly associated with poor patient-physician communication and increase the risk of patient misunderstanding and mismanaging the ocular conditions[17]. Previous studies also suggested that improving patients’ perceived level of understanding was an important factor to maximize patient satisfaction with cataract surgery[11, 12]. Patients with adequate health literacy presumably had a full understanding of potential benefits and risks of cataract surgery[17]. It is therefore not surprising that adequate health literacy was a significantly positive predictor of the discrepancy between actual and expected postoperative improvement of VF. However, adequate computer skill was not correlated with patient satisfaction, and this could be attributed to the fact that most of our patients were more than 60 years old and the majority of them had very limited computer skills, leading to a lack of statistical power for identifying potential associations.

The presence of ocular comorbidity is known to be associated with poor patient-reported outcome[8, 9, 20]. Coexisting ocular comorbidity such as glaucoma, AMD, DR and others may influence patients' vision, leading to preoperative visual impairment and increasing the risk of surgical complication. For example, studies showed a significant association between primary angle-closure glaucoma eyes and a large deviation from the target refraction after cataract surgery, which retard the outcome of the surgery[20, 21]. Stock et al. demonstrated that cataract surgery on eyes with AMD offers an increase in functional vision. However, when compared with patients without retinal pathology, AMD patients with poor preoperative visual acuity have worse visual outcomes[22]. In addition, we found that any systemic comorbidity (hypertension and diabetes) was significant correlated with the patient-reported outcome and satisfaction.

In our study, more than 50% of cataract patients had an extremely poor visual acuity before surgery and the majority of the eyes were graded less than 4.0 in LOCS III grading of nuclear opalescence and cortical cataract. LOCS III is a widely used cataract grading system, which reflects the type and severity of age-related cataract[23]. Pan's study showed that best corrected visual acuity (BCVA) was significantly correlated with the LOCS III nuclear opalescence grading but not LOCS III cortical cataract grading; and VF-14 score was significantly correlated with the BCVA and LOCS III nuclear opalescence grading of the better eye[24]. In our study, the poor surgery outcomes were significantly correlated with the low baseline VA and LOCS III cortical cataract grading (>4, the severe stage) of the surgery eye. In patients with early to moderate stages of cortical cataract, the opacities were located mainly at the periphery and the pupil area of the cortex was relatively clear. In patients with severe stage of cortical cataract, the pupil area of the cortex was opacified and the VF was obviously impaired. There is no significant correlation between patient-reported outcomes with the type of implanted IOL. However, patients with implantation of aspherical IOL are more likely satisfied with the surgery.

Strengths of our study included its prospective design and the high response rate. The data collected covered most of the major factors influencing the patient-reported outcome and patient’s satisfaction with the cataract surgery. However, there were a few limitations. First, the patients were recruited from only two tertiary referral hospitals in Guangzhou and did not reflect the general cataract patients in urban southern China. Second, preoperative expected data were compared with postoperative actual data from patients receiving first eye cataract surgery. Most of patients need bilateral cataract surgery. A further study involving second eye surgery is required to validate our results.

In conclusion, cataract patients in urban Southern China have high expectations for surgery outcomes. Patients with low level of health literacy and the presence of systemic and ocular comorbidity may need a comprehensive counseling to decrease the discrepancy regarding expected and actual outcomes and to improve patients' satisfaction.
